# A Novel Anti-B7-H3 × Anti-CD3 Bispecific Antibody with Potent Antitumor Activity

**DOI:** 10.3390/life12020157

**Published:** 2022-01-21

**Authors:** Yan Feng, Kun Xie, Yanxin Yin, Bingyu Li, Chenyu Pi, Xiaoqing Xu, Tao Huang, Jingming Zhang, Bo Wang, Hua Gu, Jianmin Fang

**Affiliations:** 1 School of Life Sciences and Technology, Tongji University, Shanghai 200092, China; fengyango12@163.com (Y.F.); kunxiebio@126.com (K.X.); yyx0301mu@126.com (Y.Y.); libingyu006@163.com (B.L.); 15221536768@163.com (C.P.); xuxiao_qing01@163.com (X.X.); huangtao@tongji.edu.cn (T.H.); zjm2020pro@outlook.com (J.Z.); wangbo15619@163.com (B.W.); 2 Biomedical Research Center, Suzhou 230031, China; 3 Shanghai Tongji Hospital, Shanghai 200065, China

**Keywords:** B7-H3, bispecific T cell engager, solid tumor, immunotherapy

## Abstract

B7-H3 plays an important role in tumor apoptosis, proliferation, adhesion, angiogenesis, invasion, migration, and evasion of immune surveillance. It is overexpressed in various human solid tumor tissues. In patients, B7-H3 overexpression correlates with advanced stages, poor clinical outcomes, and resistance to therapy. The roles of B7-H3 in tumor progression make it a potential candidate for targeted therapy. Here, we generated a mouse anti-human B7-H3 antibody and demonstrated its binding activity via Tongji University Suzhou Instituteprotein-based and cell-based assays. We then developed a novel format anti-B7-H3 × anti-CD3 bispecific antibody based on the antibody-binding fragment of the anti-B7-H3 antibody and single-chain variable fragment structure of anti-CD3 antibody (OKT3) and demonstrated that this bispecific antibody mediated potent cytotoxic activities against various B7-H3-positive tumor cell lines in vitro by improving T cell activation and proliferation. This bispecific antibody also demonstrated potent antitumor activity in humanized mice xenograft models. These results revealed that the novel anti-B7-H3 × anti-CD3 bispecific antibody has the potential to be employed in treatment of B7-H3-positive solid tumors.

## 1. Introduction

Over the past few years, the number of reports demonstrating the development and effects of therapeutic bispecific antibodies (bsAbs) has increased rapidly. Currently, various bsAbs for cancer therapy are under clinical development, and bispecific T cell engager (bsTCEs) represent the largest group. The strategy involving redirection of effector T cells to kill target cells demonstrate significant potential in cancer immunotherapy [1,2]. bsTCEs have shown potent therapeutic effects in treatment of hematologic malignancies but the benefit in treatment of solid tumors has been far less promising. The treatment of solid tumors is difficult owing to several factors such as antigen expression in critical normal tissues, immunosuppressive tumor microenvironment, disordered tumor vasculature, and less effector cells infiltration in tumor tissues. One of the hurdles is selection of a tumor-associated antigen (TAA) specifically targeting solid tumor, as expression on critical normal tissues of solid tumor TAA can lead to adverse events by on-target off-tumor T cell reactivity [3].

B7-H3 (CD276), a B7 superfamily member, was identified as a T cell co-stimulatory and co-inhibitory molecule. B7-H3 demonstrates positive regulatory functions in cytotoxic T cell proliferation, activation, and IFN-γ production in vitro [4]. Other studies have also shown the co-stimulatory role of B7-H3, which is seemingly correlated with enhanced therapy efficacy and prolonged survival [5,6,7,8]. However, most subsequent studies have demonstrated that B7-H3 is involved in T cell inhibition and is emerging as an important regulator of tumor progression [9,10,11,12,13]. B7-H3 also participates in tumor progression via a non-immunological reaction. High B7-H3 expression has been detected in multiple human solid tumor tissues such as non-small cell lung cancer [14], prostate cancer [15,16,17], pancreatic cancer [8,18], ovarian carcinoma [19], endometrial carcinoma [20], colorectal cancer [21], hepatocellular carcinoma [22], breast cancer [23], clear cell renal cell cancer [24], and head and neck cancer [25]. Besides its overexpression on tumor cells, B7-H3 is overexpressed on tumor-infiltrating dendritic cells, macrophages, monocytes, tumor-associated fibroblasts, endothelial cells, and cancer stem cells [19,24,25,26,27]. B7-H3 overexpression in tumor tissues correlates with decreased T cell infiltration, poor prognosis, increased metastasis, advanced clinical stage, and resistance to therapy [15,24,25,26,27]. Furthermore, B7-H3 expression is limited at low levels in healthy tissues [28].

Collectively, B7-H3 is a potential target for solid tumor treatment. Many therapy approaches targeting B7-H3, including monoclonal antibody (mAb), chimeric antigen receptor T cells (CAR-T), antibody-drug conjugate (ADC), and bsAb are undergoing evaluation in preclinical and clinical trials and have shown impressive therapeutic efficacy [28,29,30,31,32,33]. Although some bsTCEs targeting B7-H3 also have shown anti-tumor effects in preclinical studies, until now, only MGD009 has been studied in clinical studies. MGD009, also known as Orlotamab, is a humanized, Fc-bearing dual affinity re-targeting (DART) format molecule that recognizes B7-H3 and CD3. Treatment with MGD009 in tumor-engrafted mice showed recruitment of T cells to the tumor site and dose-dependent antitumor activity [34]. MGD009 has been studied in two clinical studies (NCT02628535, NCT03406949). Except for MGD009, most of these anti-B7-H3 bsTCEs were synthesized by linking the anti-CD3 antibody and anti-B7-H3 antibody using chemical coupling agents; thus, the size of these antibodies is large, which may negatively affect physicochemical properties such as solubility [35,36,37,38]. Some other anti-B7-H3 bsTCEs are designed as a bispecific T cell engager (BiTE) format with short half-life [39,40]. More anti-B7-H3 bsTCEs with different structures may should be pursued for B7-H3 targeting therapy.

In this study, we aimed to develop a novel format asymmetric anti-B7-H3 × anti-CD3 bispecific antibody (αB7-H3/CD3) based on antibody-binding fragment (Fab) of anti-B7-H3 mAb and single chain variable fragment (scFv) of anti-CD3 mAb (OKT3) and examine its binding activity, cytotoxic potential, and underlying mechanisms against tumor cell lines. The results demonstrated that the bsAb were able to significantly induce activation and proliferation of T cells and mediate tumor cell lysis. Furthermore, we evaluated the in vivo tumor inhibition activity of the bsAb in xenograft models of glioblatoma and ovarian carcinoma using an NCG humanized mice model. The tumors regressed and were completely eliminated upon treatment with αB7-H3/CD3.

## 2. Materials and Methods

Unless stated, experiments were performed according to manufacturer’s protocol.

### 2.1. Cell Lines

FreeStyle™ 293T cells were obtained from Thermo Fisher Scientific (Gibco, Carlsbad, CA, USA) and cultured in FreeStyle™ 293 medium (12338018, Gibco, Carlsbad, CA, USA) at 37 °C, 8% CO_2_, and 120 rpm. Human tumor cells A498, SKOV3, MDA-MB-468, A549, HepG2, BCPAP, and Raji were obtained from ATCC (Rockville, MD, USA) and stored in our lab. U-87 MG, PANC-1, MDA-MB-231, AsPC-1, and K562 were obtained from NCACC (Shanghai, China) and stored in our lab. Cells were maintained at 37 °C and 5% CO_2_. A498, U-87 MG, SKOV3, MDA-MB-468, PANC-1, A549, BCPAP, MDA-MB-231, HepG2, and AsPC-1 were cultured in DMEM (Hyclone, Waltham, MA, USA) supplemented with 100 U/mL penicillin, 100 µg/mL streptomycin (Gibco, Carlsbad, CA, USA), and 10% fetal bovine serum (FBS) (Cegrogen, Stadtallendorf, Germany). K562, and Raji were maintained in RPMI 1640 (Hyclone, Waltham, MA, USA) with 100 U/mL penicillin, 100 µg/mL streptomycin, and 10% FBS.

### 2.2. Production of Anti-B7-H3 mAbs and Bispecific Antibody

Bivalent anti-B7-H3 antibodies were produced using the hybridoma method. BALB/c mice were immunized with recombinant extracellular domain of human B7-H3 linked to His tag protein (B7-H3-ECD) (11188-H08H, SinoBiological, Beijing, China). Parental mAb against B7-H3 were screened based on the results of the protein and cell binding using ELISA and flow cytometry, respectively. The chimeric mAb (10-2#c) was generated by fusing the anti-B7-H3 antibody variable light chain (VL) to the human IgG1 light chain constant region (Cκ) and the anti-B7-H3 antibody variable heavy chain (VH) to the human IgG1 heavy chain constant region (Cγ1).

The αB7-H3/CD3 format was developed based on the heterodimeric Fc variant via the knobs-into-holes technique (KIH) [41,42]. The mutations T366S, L368A, and Y407V were introduced into CH3 domain of heavy chain hole-Fc and mutation T366W was introduced into CH3 domain of a single knob-Fc chain. Both Fc parts carried the N297A mutation to abolish Fc-mediated effector functions [43,44]. The light chain was constructed with an anti-CD3 scFv (huOKT3, GenBank: AND42858.1) fused to 10-2#c light chain C-terminus by a 15-amino acid linker (G_4_S)_3_.

### 2.3. Expression and Purification of Antibodies

DNA fragments encoding heavy and light chains were synthesized by Genewiz (Azenta Life Sciences, Suzhou, China) and cloned into the pcDNA3.1+ vector. To express antibodies, three vectors were co-transfected into FreeStyle™ 293T cells for transient expression, and supernatants were collected 7 days post-transfection. Antibodies were purified using Protein A affinity chromatography (Thermo Fisher Scientific Co., Ltd.) and then further purified using anti-flag affinity column (Flag tag was fused to C terminus of knob Fc fragment) (P2271, Beyotime, Shanghai, China). Protein samples were analyzed using SDS electrophoresis.

### 2.4. Preparation of Primary Cells

PBMCs were isolated using Ficoll-Hypaque density gradient centrifugation (Tbdscience, Tianjin, China) from healthy donors and cultured in IMDM (Hyclone, Waltham, MA, USA) supplemented with 100 U/mL penicillin, 100 U/mL streptomycin, and 10% heat-inactivated FBS [45]. Human CD3+ T cells were isolated using a negative selection human CD3 T cell isolation kit (480022, BioLegend, San Diego, CA, USA). The pre-selection and post-selection (positive and negative fractions) purities were tested using flow cytometry.

### 2.5. Cell Binding Assay

Tumor cell lines, PBMCs, and CD3+ T cells were separately incubated with serially diluted indicated concentrations (0.0001–100 µg/mL) of anti-B7-H3 mAb (10-2#c) or αB7-H3/CD3 for 1 h on ice and stained with APC-anti-human IgG (409306, BioLegend, San Diego, CA, USA) in the dark for 30 min. Cell binding activity was analyzed using flow cytometry (CytoFLEX LX, Beckman Coulter, Brea, CA, USA).

### 2.6. In Vitro Cytotoxicity Assay

Target cells (5 × 10^3^–1 × 10^4^) labeled with 2 μM calcein-AM (425201, BioLegend, San Diego, CA, USA) were co-cultured with effector cells and the indicated concentrations (0.00001–1000 ng/mL) of αB7-H3/CD3 or other antibodies (anti-CD3 mAb (317325, Biolegend, San Diego, CA, USA) or anti-B7-H3 mAb (10-2#c) or mixture) for 12–24 h at 37 °C [46]. PBMCs or CD3+ T cells from healthy donors were used as effector cells, and the effector-to-target (E:T) ratio was 10:1. After co-culture, cells were harvested and analyzed using flow cytometry. Calcein-AM+ cells were evaluated as live target cells, and the group without antibodies was analyzed as the control. Cell death X (%) was calculated as (1-percentage of live cells in X group/percentage of live cells in control group) × 100.

### 2.7. T Cell Activation Assay and Cytokine Release Assay

For the T cell activation assay, CD3+ T cells or PBMCs were co-cultured with tumor cells and 1 μg/mL of αB7-H3/CD3 or other antibodies (anti-CD3 mAb or anti-B7-H3 mAb or mixture) for 24 h in 96-well culture plates (E:T = 10:1). After that, cells were collected and stained with detection antibodies. Part of cells were stained with APC-anti-human CD4 (357408, BioLegend, San Diego, CA, USA), PC5.5-anti-human CD8 (344710, BioLegend, San Diego, CA, USA), and PE-anti-human CD69 (310906, BioLegend, San Diego, CA, USA) for 30 min in the dark. Another portion was stained with APC-anti-human CD4 (357408, BioLegend, San Diego, CA, USA) and PC5.5-anti-human CD8 (344710, BioLegend, San Diego, CA, USA), and then fixed and permeabilized prior to staining with PE-anti-human/mouse granule granzyme B (GrB) (372208, BioLegend, San Diego, CA, USA). T cell activation was determined by flow cytometry. Supernatants were collected to quantify the release of interleukin 2 (IL-2) (431804, BioLegend, San Diego, CA, USA), interleukin 6 (IL-6) (430504, BioLegend, San Diego, CA, USA), and IFN-γ (430104, BioLegend, San Diego, CA, USA) after cell culture for 48 h using ELISA kits.

### 2.8. T Cell Proliferation Assay

CFSE were used for T cell proliferation measurement [47]. U-87 MG cells (1 × 10^4^) were incubated with CFSE (423801, BioLegend, San Diego, CA, USA)-labeled purified human CD3+ T cells supplemented with 1 μg/ml αB7-H3/CD3 or other antibodies (anti-CD3 mAb and anti-B7-H3 mAb or mixture) for 5 days in 96-well plates (E:T = 10:1). Cells were collected and stained with APC-anti-human CD4 and PC5.5-anti-human CD8 antibodies. CFSE dilution was analyzed using flow cytometry.

### 2.9. Ethics Statement

NCG mice (GemPharmatech, Nanjing, China) were maintained in pathogen-free conditions. All procedures were performed under the guidelines of Directive 2010/63/EU from the European Union. The protocol was approved by the Animal Research Ethics Committee of Tongji University (No. TJLAC-018-032). Informed consent was obtained from the PBMCs donors.

### 2.10. In Vivo Xenograft Model

NCG mice (6–8 weeks old) were intravenously (i.v.) inoculated with 8 × 10^6^ huPBMCs via the tail vein approximately 2 weeks before 5 × 10^6^ U-87 MG/SKOV3 cells were subcutaneously (s.c.) transplanted into mice [48]. When tumor volume was about 150 mm^3^, U-87 MG xenograft bearing mice were randomized into six groups (no PBMC, PBS, αB7-H3/CD3 (0.2, 1, and 5 mg/kg) and anti-B7-H3 mAb 10-2#c (5 mg/kg)). SKOV3 xenograft bearing mice were randomized into four groups (no PBMC, PBS, and αB7-H3/CD3 (0.2 and 1 mg/kg)). Drugs were intraperitoneally (i.p.) injected twice per week. The bodyweight of mice and length (mm) and width (mm) of tumors were monitored every 2 or 3 days. Tumor volume (mm^3^) was calculated as Length × Width^2^ × 1/2. When the average tumor volume exceeded 1500 mm^3^, mice were euthanized.

### 2.11. Immunofluorescence Staining

Murine tumor tissues sections were fixed in 4% paraformaldehyde for 20 min at 20 °C and blocked for 10 min. Sections were then stained with anti-CD3 antibody (GB111337, Servicebio, Wuhan, China) overnight at 4 °C, Cy3 conjugated secondary antibody (GB21303, Servicebio, Wuhan, China) for 1 h, and DAPI (G1012, Servicebio, Wuhan, China) for 15 min in the dark. Images were captured with a fluorescence microscope.

### 2.12. Statistical Analysis

Statistical analyses were performed by GraphPad Prism 7.0. Statistical significance between groups was calculated using an unpaired two-tailed *t*-test or two-way/one-way analysis of variance (ANOVA). For all figures, NS, non-significant; **** *p* < 0.0001; *** *p* < 0.001; ** *p* < 0.01; * *p* < 0.05. Data are shown as means ± SD.

## 3. Results

### 3.1. Analysis of B7-H3 Expression and Generation of Anti-B7-H3 Antibody

**Figure 1:**
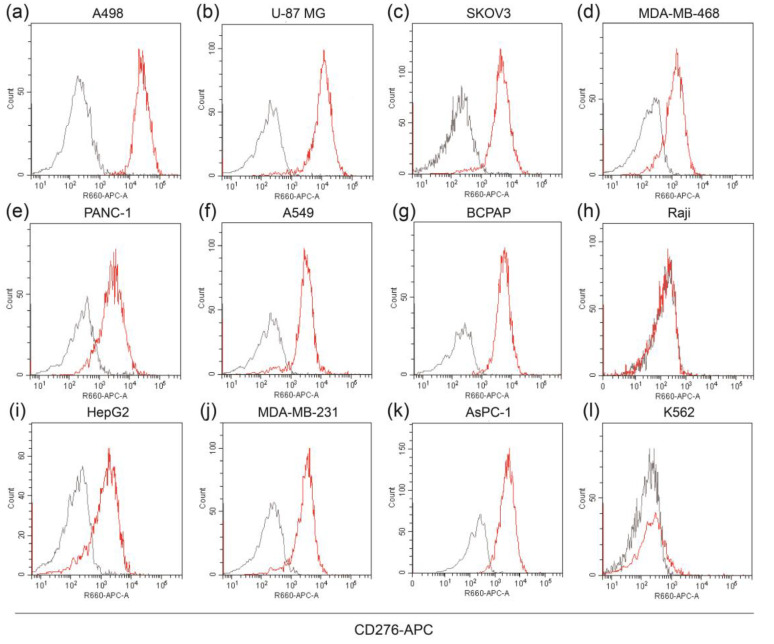
B7-H3 is highly expressed on human solid tumor cell lines. (**a**–**l**) Expression of B7-H3 on cell lines (A498, U-87 MG, SKOV3, MDA-MB-468, PANC-1, A549, BCPAP, Raji, HepG2, MDA-MB-231, AsPC-1, and K562) was detected by flow cytometry. Gray histogram, isotype control; red histogram, APC-conjugated anti-human CD276 (10 μg/mL). MFI values (control/anti-human CD276) of these cell lines were, respectively, 160/25,090, 62/10,733, 62/4317, 57/1240, 134/2472, 67/2918, 60/3315, 4.5/15, 71/1262, 63/2966, 60/2911, and 26/84. MFI, median fluorescence intensity.

B7-H3 is highly expressed in various human carcinomas. To analyze B7-H3 expression on cell lines surface, we characterized human tumor cell lines from different tumor systems using flow cytometry. As shown in Figure 1a–l, most of the solid tumor cell lines showed a high expression of B7-H3 (A498, U-87 MG, SKOV3, A549, BCPAP, and MDA-MB-231); however, the hematoma tumor cell lines (K562 and Raji) showed low B7-H3 expression. On the surface of Raji cells, B7-H3 expression is almost negative.

**Figure 2:**
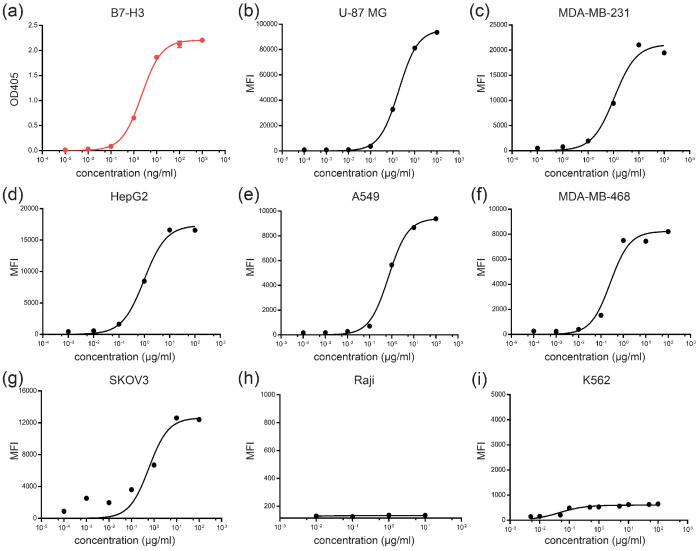
Anti-B7-H3 antibody 10-2#c shows high binding activity to B7-H3 and cell lines with high B7-H3 expression. (**a**) The binding of 10-2#c to human B7-H3 reconstituted protein was evaluated by ELISA (*n* = 3), EC_50_ = 2.216 ng/ml; (**b**–**i**) The binding of 10-2# to cell lines (U-87 MG, MDA-MB-231, HepG2, A549, MDA-MB-468, SKOV3, Raji, and K562) was detected by flow cytometry. MFI, median fluorescence intensity. MFI values (control/maximum concentration of 10-2#c) of these cell lines were, respectively, 834/93,630, 366/19,477, 353/16,980, 144/9385, 245/8215, 250/12,400, 125/136, and 140/646.

To study B7-H3-targeting therapy, we generated anti-B7-H3 monoclonal antibodies by immunizing BALB/c mice with B7-H3 ECD-His and selected antibody 10-2# as the candidate for further studies based on the results of the binding assay. We then generated the chimeric antibody 10-2#c by fusing 10-2# VL to human Cκ and 10-2# VH to human Cγ1. The 10-2#c bound to human B7-H3-ECD protein with a half-maximal effective concentration (EC_50_) value of 2.216 ng/ml (Figure 2a). The antibody showed high binding activity to tumor cells showing high expression of B7-H3 (Figure 2b–i).

### 3.2. Generation and Characterization of αB7-H3/CD3

**Figure 3:**
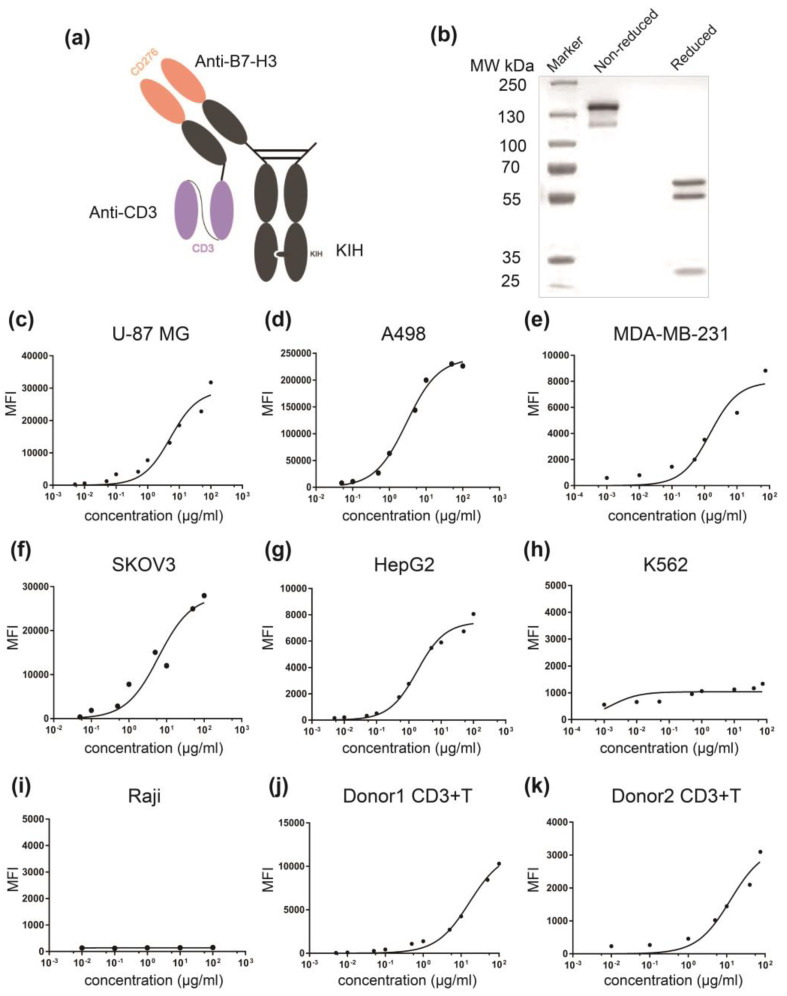
Generation and characterization of αB7-H3/CD3 bispecific antibody. (**a**) An illustrative representation of the αB7-H3/CD3 format. The format comprised an anti-CD3 scFv fused to light chain of a monovalent anti B7-H3 via a (G_4_S)_3_ linker; (**b**) Coomassie blue-stained SDS-PAGE analysis of purified αB7-H3/CD3 containing three chains with the following molecular weights: 57, 55, and 28 kDa; (**c**–**k**) αB7-H3/CD3 bispecific antibody dose-dependently binds to B7-H3+ tumor cells and CD3+ T cells, evaluated using flow cytometry. MFI values (control/maximum concentration of 10-2#c) of these cell lines were, respectively, 205/31,800, 7500/226,444, 480/5700, 350/27,950, 150/8065, 480/1550, 125/155, 50/10,300, and 195/3100.

To develop a bsAb for B7-H3-targeting, we constructed a bsTCE by linking scFv of anti-CD3 mAb (OKT3) to C-terminus of monovalent, single-arm anti-B7-H3 mAb light chain (Figure 3a). Purity and correct molecular weights of αB7-H3/CD3 were detected by non-reduced SDS-PAGE, and reduced SDS-PAGE showed three bands of αB7-H3/CD3: a 55 kDa light chain, a 57 kDa hole chain, and a 28 kDa knob chain (Figure 3b). Flow cytometry revealed the high binding activity of αB7-H3/CD3 to cell lines showing high expression of B7-H3 (U-87 MG, A498, SKOV3, and MDA-MB-231) but not to cell lines showing low expression of B7-H3 (K562 and Raji) (Figure 3c–i). The antibody could dose-dependently bind to human CD3+ T cells (Figure 3j–k). Therefore, αB7-H3/CD3 demonstrated the ability to bind specifically to both B7-H3 and CD3 on the cell surface.

### 3.3. αB7-H3/CD3 Mediates T Cell Cytotoxicity against B7-H3-Overexpressing Cells In Vitro

**Figure 4:**
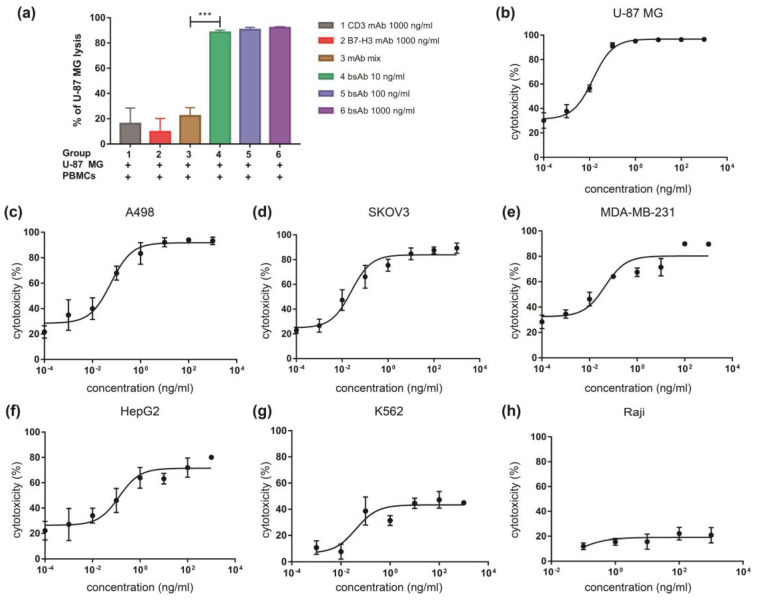
αB7-H3/CD3 mediates T cell cytotoxicity against B7-H3 expressing tumor cells in vitro. (**a**) Cytotoxic activity of PBMCs in U-87 MG cells mediated by αB7-H3/CD3 or other antibodies was measured by flow cytometry. *n* = 3, *p*-value for mAb mix vs. bsAb: *** *p* < 0.001; (**b**–**h**) αB7-H3/CD3 demonstrated cytotoxic activity against multiple tumor cell lines (U-87 MG, A498, SKOV3, MDA-MB-231, HepG2, K562, and Raji) with various levels of B7-H3 expression, E:T = 10:1. *n* = 3, mAb mix, anti-CD3 and anti-B7-H3 mAb mixture; bsAb, αB7-H3/CD3.

We then evaluated whether engagement of B7-H3 high expression cells and T cells mediated by αB7-H3/CD3 can trigger tumor cell lysis. The potency of cytotoxic effects was first assessed against the B7-H3-high expression cell line U-87 MG. Results showed remarkable αB7-H3/CD3-mediated T cell cytotoxicity against U-87 MG cells, and U-87 MG cells were lysed in dose-dependent manner (Figure 4a,b). The anti-B7-H3 or/and anti-CD3 mAb could mediate the cytotoxicity activity leading to tumor cell death, but the effect was limited (Figure 4a).

Next, we tested the cytotoxic activities of αB7-H3/CD3 against the B7-H3 low-expressing cell lines (K562 and Raji) and multiple cell lines (A498, SKOV3, MDA-MB-231, and HepG2) with various levels of surface B7-H3 expression. Data showed that αB7-H3/CD3 led to minimal cell lysis in B7-H3 low expression tumor cell lines. However, it demonstrated a highly cytotoxic effect resulting in cell death in multiple tumor cell lines with high levels of B7-H3 (Figure 4b–h). These results suggested that αB7-H3/CD3-mediated cytotoxicity was B7-H3-specific, and its potency depended on B7-H3 expression.

### 3.4. αB7-H3/CD3 Mediates T Cell Activation and Promotes T Cell Proliferation

**Figure 5:**
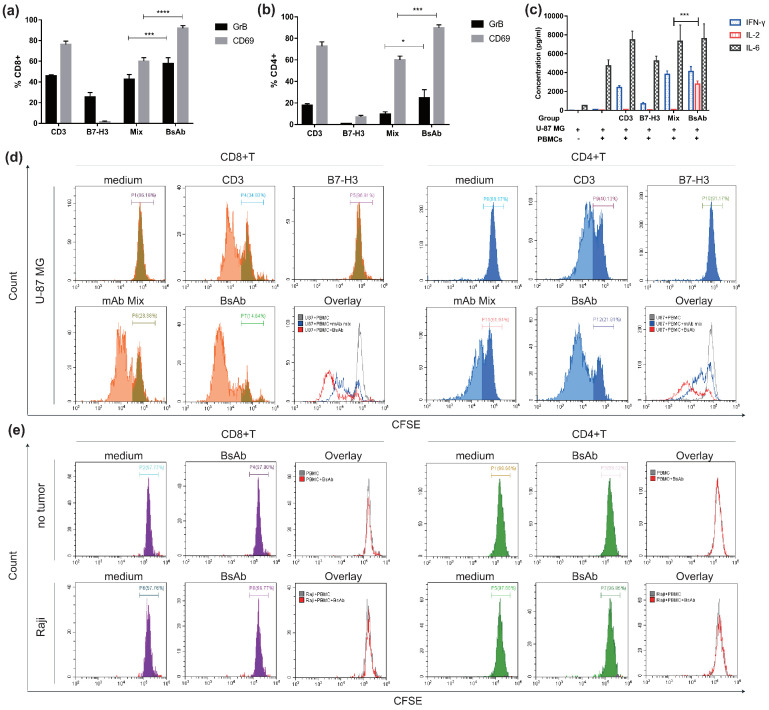
αB7-H3/CD3 induces T cell activation and proliferation in vitro. U-87 MG and PBMCs were co-cultured with indicated antibodies as described in methods. T cell activation markers (CD69 and GrB) and T cell proliferation were analyzed in CD8+ and CD4+ subsets by flow cytometry. The profile of cytokines released by PBMCs was quantified using ELISA. (**a**,**b**) Percentage of CD69 and GrB-positive cells in CD4+ or CD8+ T cell subsets. *n* = 3; (**c**) Secretion of cytokines by PBMCs induced by the indicated antibodies. *n* = 3; (**d**,**e**) T cell proliferation was measured using CFSE dilutions. Groups in which U-87 MG was replaced with no tumor or Raji (**e**). Representative histograms of CFSE-labeled T cells in CD8+ (**left**) and CD4+ (**right**) T cell subsets are shown. CD3, anti-CD3 mAb; B7-H3, anti-B7-H3 mAb; mAb mix, anti-CD3 and anti-B7-H3 mAb mixture; bsAb, αB7-H3/CD3. **** *p* < 0.0001; *** *p* < 0.001; * *p* < 0.05.

The potency of αB7-H3/CD3 to mediate T cell activation was examined by activation markers (CD69 and cytotoxic granule granzyme B (GrB) expression and cytokine production. Anti-CD3 mAb was used as a positive control. Additionally, since B7-H3 is an immune checkpoint molecule, we wanted to explore whether this anti-B7-H3 mAb could play the role of immune checkpoint blockade. As shown in Figure 5a,b, αB7-H3/CD3 activated CD8+ and CD4+ T cells to a similar extent as anti-CD3 mAb. Anti-B7-H3 mAb had little effect on T cell activation. As shown in Figure 5c, αB7-H3/CD3 mediated significantly more abundant production of IL-2 compared to anti-CD3 mAb or/and anti-B7-H3 mAb. Figure 5d showed that the anti-B7-H3 mAb did not induce T cell proliferation and demonstrated that αB7-H3/CD3 had a stronger effect on induction of both CD8+ (left) and CD4+ (right) T cell proliferation compared to anti-CD3 mAb and mAb mix. Additionally, when we replaced U-87 MG with no tumor or Raji, αB7-H3/CD3 did not induce T cell proliferation, which indicated that this effect was associated with the expression of B7-H3 (Figure 5e). These results confirmed that αB7-H3/CD3-mediated activity was accompanied by T cell expansion and activation, and its effect on T cells was B7-H3-dependent. Moreover, αB7-H3/CD3 had a stronger effect on the activation and proliferation of CD8+ T cells than CD4 + T cells.

### 3.5. αB7-H3/CD3 Is Potent in Tumor Growth Inhibition In Vivo

**Figure 6:**
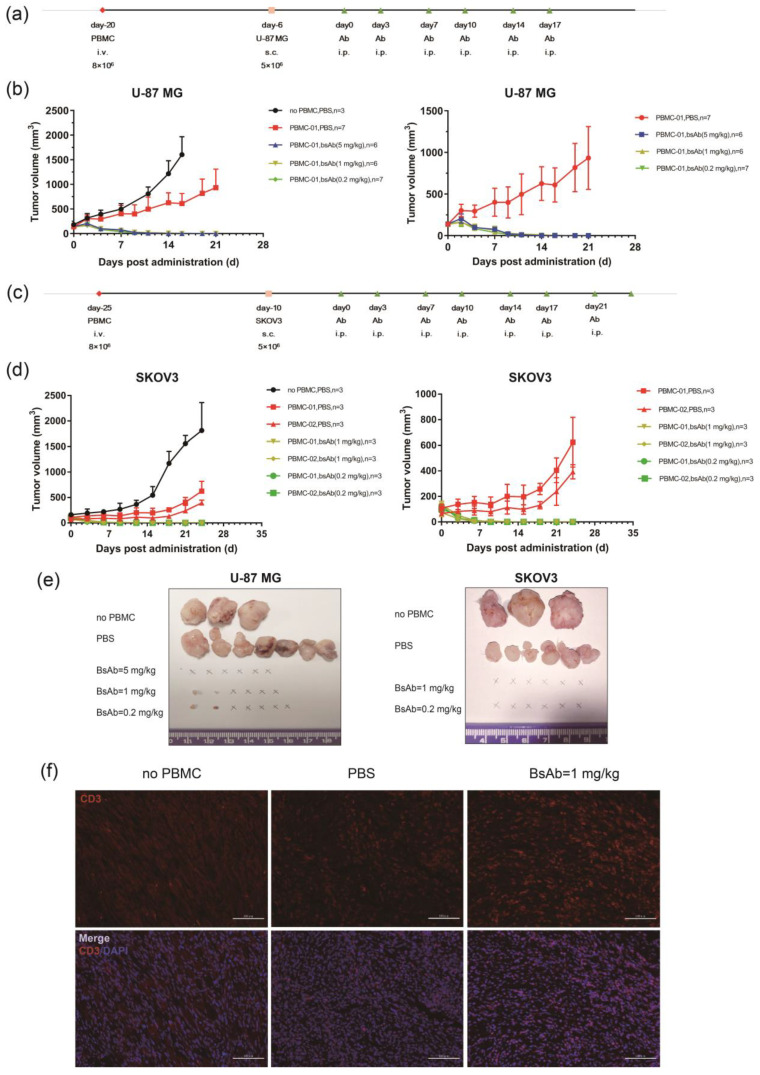
αB7-H3/CD3 inhibits tumor growth in xenograft models using human PBMC-engrafted NCG mouse models. (**a**,**c**) Schematic map of the development of U-87 MG (**a**) and SKOV3 (**c**) xenograft model. U-87 MG or SKOV3 were subcutaneously implanted into NCG mice engrafted with human PBMCs. Mice were treated with PBS and doses of αB7-H3/CD3 twice a week post tumor development; (**b**,**d**) average tumor growth curve of U-87 MG (**b**) and SKOV3 (**d**) xenografts for every group. The tumor volume was plotted against the time in days following antibody administration. The number of mice is shown in figures; (**e**) images of individual xenograft tumors at the end of experiments; (**f**) immunofluorescence staining analysis of CD3+ T cells (red) in U-87 MG tumors. Scale bar, 100 μm. bsAb, αB7-H3/CD3.

To determine the antitumor efficacy of αB7-H3/CD3 in vivo, the glioblastoma cell line U-87 MG and ovarian carcinoma cell line SKOV3 were used to establish xenograft models in human PBMC-reconstituted mice. U-87 MG/SKOV3 cells (5 × 10^6^) were implanted subcutaneously into the huPBMC-NCG mice. Mice demonstrating tumor development (150 mm^3^) were treated with doses of αB7-H3/CD3, PBS, or/and anti-B7-H3 mAb (i.p., 2×/wk × 3 weeks). Tumor volume was evaluated (Figure 6a,c). Figure 6 summarizes the results of these experiments. The inhibitory effect of αB7-H3/CD3 treatment on tumor growth was significant. As shown in Figure 6b, U-87 MG xenografts were completely eliminated after either three or five injections of 5mg/kg of αB7-H3/CD3 (i.p.), while the anti-B7-H3 mAb did not suppress tumor growth (Figure S1). In the groups treated with 0.2 and 1 mg/kg αB7-H3/CD3, complete inhibition of xenografts was observed in most mice (5/7 of 0.2 mg/kg group, 4/6 of 1 mg/kg group), with a few animals showing small nodules (2/7 of 0.2 mg/kg group, 2/6 of 1 mg/kg group) (Figure 6b,e). In the SKOV3 xenograft model, PBMCs derived from two donors were used to reconstruct the mouse immune system. According to the tumor growth curve, tumors were eradicated in all αB7-H3/CD3-treated mice regardless of the source of PBMCs (Figure 6d,e). Immunofluorescence staining analysis of isolated U-87 MG tumors showed more T cell infiltration in αB7-H3/CD3-treated mice, indicating that αB7-H3/CD3 effectively recruited and maintained T cells (Figure 6f). These experiments showed that αB7-H3/CD3 effectively recruited T cells into tumor sites and mediated antitumor activity in murine xenograft models.

## 4. Discussion

Many bsTCEs targeting classical solid tumor antigens, such as HER2, EGFRvIII, PSMA, EpCAM, and CEA, are being explored in clinical practice, and multiple other TAAs are currently pursued in preclinical studies [3]. B7-H3 is a co-inhibitory molecule that overexpressed in various solid tumor tissues and limited in normal tissues, making it an attractive target for bsAb therapy. Here, we described the generation of a novel bsTCE specific to T cells and B7-H3 positive tumors and demonstrated the potential application.

Structure is important for the development of bsAbs. Santich et al. demonstrated that a symmetric dual bivalent bsAb format (IgG-[L]-scFv (“2 + 2”): anti-CD3 scFv fused to the C terminus of each antitumor IgG light chain) is more potent than other common bsAb designs (such as BiTE and IgG heterodimer) and IgG-[L]-scFv heterodimers with different combinations of valency and inter-domain spatial configurations [49]. They revealed the beneficial effects of cis-configurations and inter-domain spacing. The asymmetric format developed in our study is similar to one described in their study referred to as “1 + 1C”. “1 + 1C” had one antitumor domain replaced and one anti CD3 domain removed from the same side (cis) of “2 + 2” format. The monovalent format is not good as “2 + 2” format in terms of anti-tumor efficacy but closely resembles native antibody in size and has inferior T cell binding. We also generated another format in which anti-CD3 scFv is fused to the N terminus of the antitumor light chain and demonstrated that the format has superior T cell binding compared to anti-CD3 scFv fused to C terminus format (Figure S2). Several studies have shown correlation between CD3 affinity and bsTCE distribution, with higher CD3 affinity shifting bsTCE away from tumor to T-cell–rich tissues [50,51], implying that the format in our study could mitigate on-target off-tumor toxicities.

In addition, asymmetric structure is complicated to manufacture due to the mispairing of heavy and light chains. Here, hetero-dimerization of the heavy chains is achieved by KIH technology, and there is no light chain pairing as knob Fc is a single fragment. Moreover, the desired end-product can be isolated by designing purification strategies based on sequential affinity chromatography or size differences. Even so, the formation of hole–hole homo-dimers and other impurities reduced the yield of desired end-product. It is necessary to optimize the amino acid sequences or use other strategies to promote the hetero-dimerization of the heavy chains, and the humanization of anti-B7-H3 domain is also essential in subsequent researches.

We confirmed that B7-H3 was highly expressed on surface of most solid tumor cell lines and less expressed on hematoma cell lines. In vitro cytotoxicity analysis of these cell lines showed that the αB7-H3/CD3-mediated co-engagement of B7-H3 and CD3 resulted in potent lysis of tumor cells with high B7-H3 expression. These indicated a relationship between expression levels of B7-H3 and αB7-H3/CD3 mediated T cell antitumor activity. Our research also suggested that αB7-H3/CD3 mediated antitumor activity was accompanied by T cell proliferation and activation and this effect is B7-H3-dependent, and αB7-H3/CD3 has a stronger effect on the activation and proliferation of CD8+ T cells compared to CD4+ T cells. A previous work indicates a role of IL-2 in promoting differentiation of CD8+ effector and memory T cells [52]. In this study, αB7-H3/CD3 significantly potentiated production of IL-2, which may contribute to enduring the immune responses.

The in vivo tumor growth inhibition activity of antibodies was influenced by the mouse model, the timing of treatment, and the means of administration. Some preclinical studies examined the bsTCEs efficacy using a co-implantation model in which T cells and tumor cell mixtures were implanted into immune-deficient mice. Although this model can shorten the experimental period, it is difficult to accurately evaluate the antitumor efficacy of antibodies because of the lack of tumor development and T cell infiltration [53]. In addition, PBMC infusion could inhibit the onset of tumor and PBMCs from different donors may have vary influence on tumor establish (Figure 6). Thus, PBMCs derived from patients may be better to establish the humanized immune system and the immunosuppressive microenvironment. In our study, PBMCs from healthy donors were inoculated into immune deficient mice two weeks before tumor implantation to develop a humanized murine model of the immune system. αB7-H3/CD3 was administered when the tumor volume reached approximately 150 mm^3^. We observed the inhibition of established tumors and increase of T cells infiltration mediated by αB7-H3/CD3. The results demonstrated that αB7-H3/CD3 effectively recruit T cells into tumor tissue to suppress tumor growth of B7-H3-positive xenograft. We also observed the relief of GVHD symptoms in mice treated with αB7-H3/CD3, which was manifested in improvement of mental state, dermatitis symptoms, and liver and kidney damage (Figure S3). This indicated that αB7-H3/CD3 did not bring additional toxicity to mice.

We did not observe antitumor activity in vivo following treatment with anti-B7-H3 mAb alone, consistently with in vitro results. These results indicated that the ADCC effect mediated by anti-B7-H3 mAb was insufficient to inhibit tumor growth, and this mAb did not play a role as inhibitor of the co-inhibitory molecule B7-H3. Therefore, the mechanism of αB7-H3/CD3 action may only attribute to redirect T cells to target cells that trigger T cell activation.

In summary, we describe a novel format for B7-H3-specific, bispecific antibodies. The bsAb mediated strong in vitro antitumor activity against B7-H3 high-expression tumor cells derived from a wide range of tumor subtypes and may cause less on-target off-tumor toxicities. The αB7-H3/CD3 demonstrated potent suppression of tumor growth in human PBMC-engrafted mice. Our study supports the hypothesis that the novel anti-B7-H3 × anti-CD3 bsAb is a potential therapeutic strategy for treating B7-H3-positive solid tumors.

## Supplementary Materials

The following supporting information can be downloaded at: https://www.mdpi.com/article/10.3390/life12020157/s1, Figure S1: Individual tumor growth curve of U-87 MG xenografts of indicated treatments; Figure S2: Comparison of CD3 binding activity of two formats anti-B7-H3/CD3 bsAb; Figure S3: αB7-H3/CD3 elicited no toxicities to liver and kidney.
