# Supplementary material for: A Novel Anti-B7-H3 × Anti-CD3 Bispecific Antibody with Potent Antitumor Activity
Source: Life (Basel). 2022 Jan 21;12(2):157. doi: 10.3390/life12020157 (PMC8879513; doi:10.3390/life12020157)
Supplement: Supplementary file 1 [file life-12-00157-s001.zip › life-1539023-supplementary.pdf]

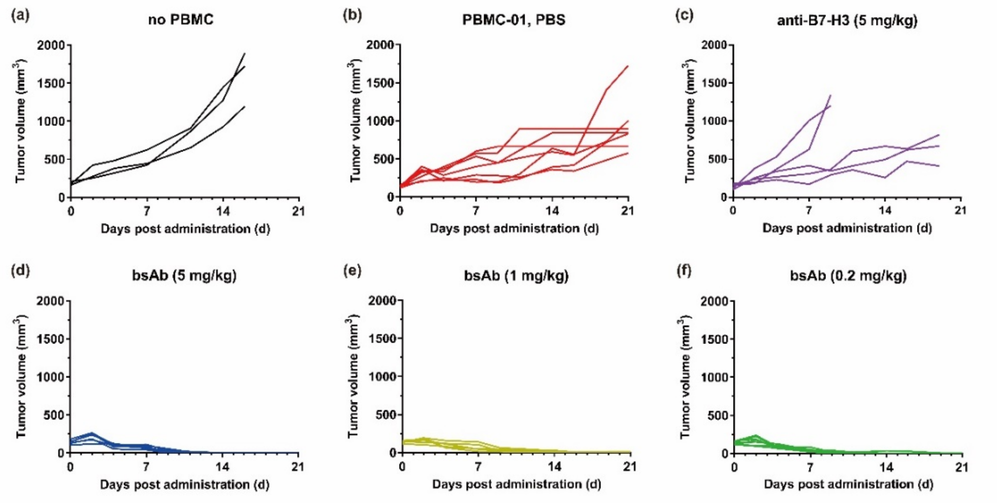

Figure S1. Individual tumor growth curve of U-87 MG xenografts of indicated treatments. (a) Individual tumor growth curve of U-87 MG xenografts in mice without PBMC-engrafted. (b-f) Individual tumor growth curve of U-87 MG xenografts in mice with PBMC-engrafted following treatment with PBS (b), anti-B7-H3 mAb (5 mg/kg) (c), anti-B7-H3/CD3 bsAb (5 mg/kg (d), 1 mg/kg (e), 0.2 mg/kg (f)). Each line represents a single mice.

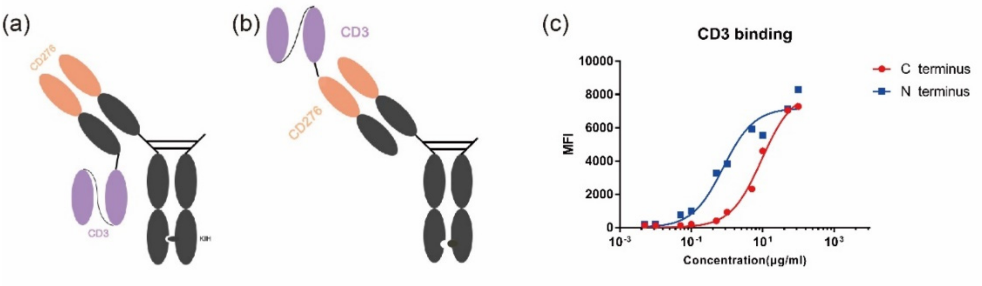

Figure S2. Comparison of CD3 binding activity of two formats anti-B7-H3/CD3 bsAb. (a and b) Schematic of C terminus format (a) and N terminus format (b), in which anti-CD3 scFv was fused to C terminus (a) or N terminus (b) of a monovalent anti-B7-H3 light chain via $(G_4S)_3$ linker. (c) Representative cell binding activity of each bsAb against Jurkat cells, measured by flow cytometry.

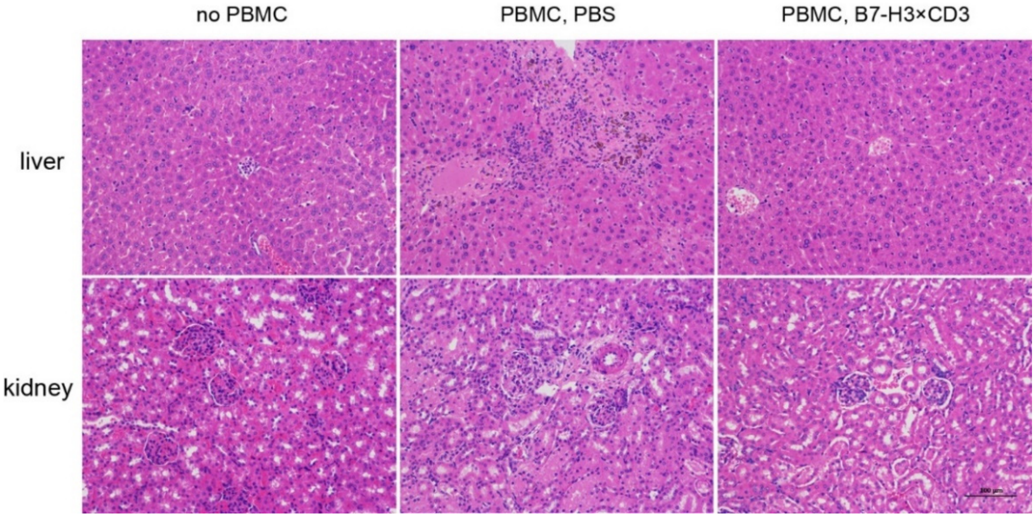

Figure S3. αB7-H3/CD3 elicited no toxicities to liver and kidney. Tissues were collected and stained with haematoxylin and eosin. Scale bar indicates 100μm.
